# Multiple Evolutionarily Conserved Domains of Cap2 Are Required for Promoter Recruitment and Iron Homeostasis Gene Regulation

**DOI:** 10.1128/mSphere.00370-18

**Published:** 2018-08-01

**Authors:** Manjit Kumar Srivastav, Neha Agarwal, Krishnamurthy Natarajan

**Affiliations:** a School of Life Sciences, Jawaharlal Nehru University, New Delhi, India; Carnegie Mellon University

**Keywords:** *Candida albicans*, Cap2/Hap43, iron homeostasis, transcriptional regulation

## Abstract

Iron is required for growth and metabolism by virtually all organisms. The human fungal pathogen Candida albicans has evolved multiple strategies to acquire iron. The Cap2/Hap43 transcriptional regulator, essential for robust virulence of C. albicans, controls iron homeostasis gene expression by promoter binding and repression of iron utilization genes. The expression of iron uptake genes is also dependent on Cap2, although Cap2 was not recruited to its promoters. Cap2, bearing the conserved bipartite HAP4L-bZIP domain, also contains multiple blocks of amino acids that form the highly conserved carboxyl-terminal region. In this study, we sought to identify the requirements of the different domains for Cap2 function. We constructed a series of mutants bearing either point mutations or deletions in the conserved domains and examined Cap2 activity. Deletion of the highly conserved extreme C-terminal region did not impair expression of Cap2 mutant protein but impaired cell growth and expression of iron homeostasis genes under iron-depleted conditions. Mutations in the amino-terminal HAP4L and basic leucine zipper (bZIP) domains also impaired growth and gene expression. Furthermore, chromatin immunoprecipitation (ChIP) assays showed that the HAP4L domain and the bZIP domain are both essential for Cap2 recruitment to *ACO1* and *CYC1* promoters. Unexpectedly, the C-terminal conserved region was also essential for Cap2 promoter recruitment. Thus, our results suggest that Cap2 employs multiple evolutionarily conserved domains, including the C-terminal domain for its transcriptional activity.

**IMPORTANCE** Iron is an essential micronutrient for living cells. Candida albicans, the predominant human fungal pathogen, thrives under diverse environments with vastly different iron levels in the mammalian host. Therefore, to tightly control iron homeostasis, C. albicans has evolved a set of transcriptional regulators that cooperate to either upregulate or downregulate transcription of iron uptake genes or iron utilization genes. Cap2/Hap43, a critical transcriptional regulator, contains multiple conserved protein domains. In this study, we carried out mutational analyses to identify the functional roles of the conserved protein domains in Cap2. Our results show that the bZIP, HAP4L, and the C-terminal domain are each required for Cap2 transcriptional activity. Thus, Cap2 employs multiple, disparate protein domains for regulation of iron homeostasis in C. albicans.

## Abstract (precis)

Iron is an essential micronutrient for living cells. Candida albicans, the predominant human fungal pathogen, thrives under diverse environments with vastly different iron levels in the mammalian host. Therefore, to tightly control iron homeostasis, C. albicans has evolved a set of transcriptional regulators that cooperate to either upregulate or downregulate transcription of iron uptake genes or iron utilization genes. Cap2/Hap43, a critical transcriptional regulator, contains multiple conserved protein domains. In this study, we carried out mutational analyses to identify the functional roles of the conserved protein domains in Cap2. Our results show that the bZIP, HAP4L, and the C-terminal domain are each required for Cap2 transcriptional activity. Thus, Cap2 employs multiple, disparate protein domains for regulation of iron homeostasis in C. albicans.

## INTRODUCTION

The eukaryotic CCAAT-binding complex (CBC) is an evolutionary conserved heteromeric transcriptional regulatory complex present from yeasts to humans (1, 2). The plant and mammalian CBCs are formed of triads of subunits composed of NFY-A, -B, and -C referred to as the NFY complex (3, 4). The budding yeast *Saccharomyces cerevisiae* CBC represented by the HAP complex is composed of Hap2, -3, -5, and -4 (3, 4). Whereas the budding yeast HAP complex regulates respiratory pathway gene expression, other fungal CBCs are shown to be involved in diverse regulatory pathways such as regulation of primary and secondary metabolism, iron homeostasis, oxidative stress response, azole drug resistance, as well as fungal pathogenesis (3, 4).

The CBC in Candida albicans, composed of Hap2-Hap3-Hap5 and the Hap4-like subunit Cap2/Hap43, is an essential regulator of iron homeostasis (5–7). Cap2 contains a 17-amino-acid Hap4-like domain and yeast AP-1 (YAP)-like basic leucine zipper domain (bZIP domain) in its N terminus that is conserved in Cap2 orthologs in *Candida*, Schizosaccharomyces pombe, and *Aspergillus* spp. but not in Saccharomyces cerevisiae (6). Deletion of *CAP2* impaired C. albicans growth in iron-depleted media and attenuated virulence of C. albicans (6, 7). Transcriptome analysis showed that ~929 genes (~15.8% of the open reading frames [ORFs]) are under Cap2 control (6), indicating a large genome-wide dependence for Cap2. Chromatin immunoprecipitation with microarray technology (ChIP-chip) analyses revealed that Cap2 was exclusively recruited to promoters of iron utilization genes under iron deprivation conditions (8), indicating that Cap2 functions as a transcriptional repressor under iron starvation conditions. Similar repressive function has been shown for HapX, the Cap2 ortholog, in Cryptococcus neoformans, S. pombe, and Aspergillus nidulans under iron starvation conditions (9–11), and it also controls expression of vacuolar iron storage genes under high iron conditions in A. nidulans (12).

Mutations in the HAP4L and bZIP domains inactivated Cap2 activity (6). Moreover, Cap2 and its orthologs from the genomes of other *Candida* clade organisms and filamentous fungi contain multiple evolutionarily conserved C-terminal regions. However, it was unclear how these specific domains contributed to Cap2 function. In this report, we show that mutations within the evolutionarily conserved N-terminal and C-terminal domains in Cap2 impaired C. albicans resistance to iron deprivation and iron homeostasis gene expression. Mutations in both the HAP4L and bZIP domains also impaired Cap2 occupancy to the *ACO1* and *CYC1* target promoters *in vivo*. Surprisingly, deletion of the conserved C-terminal 54 amino acids also impaired Cap2 promoter occupancy, thus indicating the requirement of multiple domains for Cap2 transcriptional activity *in vivo*.

## RESULTS

### Cap2 C-terminal sequence is conserved in fungal genomes and is required for C. albicans resistance to iron deprivation.

The Cap2 protein contains the bipartite HAP4L and bZIP domains in the amino-terminal region (6). Furthermore, motif search in the SMART database revealed multiple low-complexity sequences characterized by Ser-rich or Cys-rich regions (6). The Cap2 ortholog in the filamentous *Aspergillus* spp., HapX, also contains the bZIP and HAP4L domains and multiple low-complexity sequences, including Cys-rich regions (4, 6, 12, 13). To better understand the evolutionary conservation of the carboxyl-terminal sequences, we carried out multiple-sequence alignment (MSA) with Cap2 orthologs from 12 closely related fungal genomes, including Cap2 from the *Candida* CTG clade (14), HapX from filamentous fungi (Aspergillus nidulans, A. fumigatus, and A. niger), Php4 from S. pombe, and Hap4 from S. cerevisiae. The resulting phylogenetic tree showed that the Cap2 sequences from the *Candida* clade and the HapX sequences formed separate clusters, with Php4 and Hap4 forming an outgroup (see Fig. S1 at https://www.jnu.ac.in/Faculty/natarajan/data.htm). MSA revealed that in addition to the highly conserved bipartite HAP4L and bZIP domains identified previously (6), several blocks of amino acid residues showed very high conservation in the Cap2 orthologs within the *Candida* CTG clade genomes and with HapX sequences (see Fig. S2 at https://www.jnu.ac.in/Faculty/natarajan/data.htm). Notably, the C-terminal regions of all sequences, barring Hap4 and Php4, contain a highly conserved sequence corresponding to residues 580 to 634 of the Cap2 sequence and bear a solitary Cys residue at position 584. Additionally, three Cys-rich motifs, viz., Cys-X_2_-Cys-X_4_-Cys-X-Cys (termed CRR-A), Cys-Gly-Phe-Cys-X_3_-Thr-Pro-Cys-Ile/Val-Cys (CRR-B), and Cys-X_3_-P-X_2_-Cys-X_2_-Cys-X_2_-D-P-X_2_-Thr-Leu-Phe-Cys (CRR-C) are also conserved (see Fig. S2 at https://www.jnu.ac.in/Faculty/natarajan/data.htm). The Cap2 sequences from *Candida* clade organisms also contained a Ser-rich region comprising the SPX_2_SX_1_YSVQQISPAPSX_1_DSPP sequence spanning residues 209 to 230 as shown in the multiple-sequence alignment (see Fig. S2 at https://www.jnu.ac.in/Faculty/natarajan/data.htm). Thus, in addition to the amino-terminal HAP4L and bZIP domains, Cap2 has a highly conserved C-terminal region, but its function was not understood.

**FIG 1:**
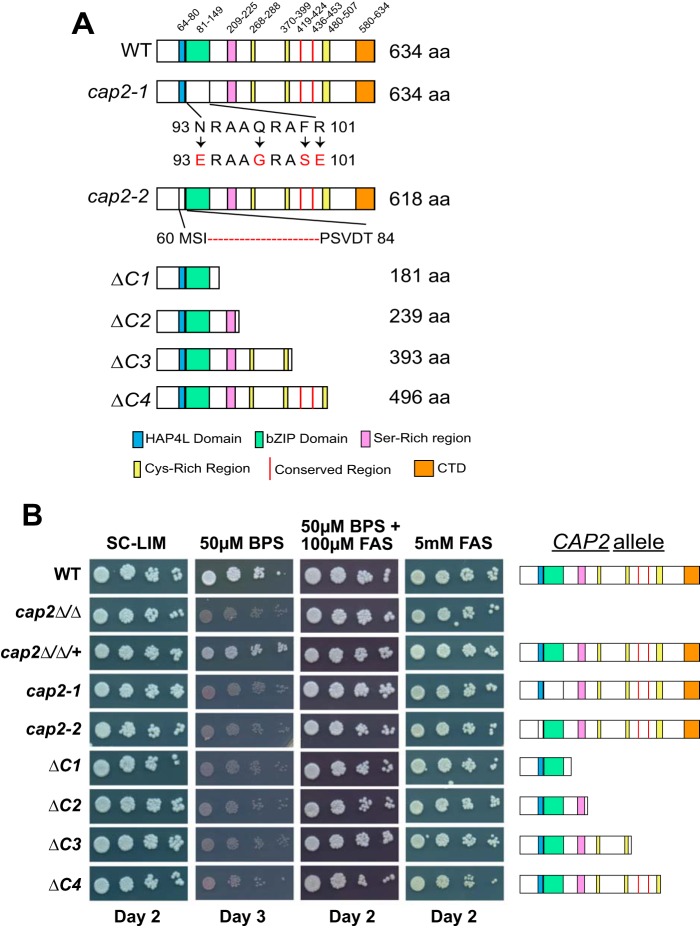
Cap2 evolutionary conserved domains are essential for growth under iron-deprived conditions. (A) Schematic diagram of wild-type (WT) and mutant derivatives of Cap2. aa, amino acids; CTD, C-terminal domain. (B) Growth phenotype of the wild-type strain, and strains bearing the various N-terminal and C-terminal mutations in Cap2. The strains were precultured in SC-LIM liquid medium, serially diluted and spotted on SC-LIM alone or on SC-LIM plus BPS, SC-LIM plus BPS and FAS, or SC-LIM plus FAS.

To investigate the requirement of the Cap2 C-terminal region, we constructed *CAP2* mutant plasmids bearing a series of C-terminal truncations termed *ΔC1*, *ΔC2*, *ΔC3*, and *ΔC4* (Fig. 1A). The *CAP2* mutant plasmids, cloned under the native *CAP2* promoter, were integrated at the *RPS1* locus, and the growth of the mutants along with control strains, including the parental *CAP2* strain, were tested under iron-depleted, iron-replete, or excess iron conditions. In addition, the bZIP and HAP4L mutants, *cap2-1* and *cap2-2* strains, respectively, were also included (6). The phenotype data showed that, as expected, the *cap2Δ/Δ* mutant could not grow in iron-depleted medium compared to the control *CAP2* strain (Fig. 1B). Adding iron in the form of ferrous ammonium sulfate (FAS) to medium containing bathophenanthrolinedisulfonic acid (BPS) rescued the growth defect of the *cap2Δ/Δ* mutant (Fig. 1B) as reported previously (6, 7). Reintegration of *CAP2* in the *cap2Δ/Δ* mutant also restored the growth under iron-depleted conditions (Fig. 1B). As we reported previously, the *cap2-1* (bZIP domain mutant) and *cap2-2* (*HAP4LΔ* mutant) strains showed severe growth defects in BPS-containing medium (Fig. 1B) (6). Interestingly, the growth of all four strains with C-terminally truncated mutant plasmids *ΔC1*, *ΔC2*, *ΔC3*, and *ΔC4* was impaired under iron-depleted conditions, and growth was restored upon iron supplementation (Fig. 1B). However, the wild-type strain and none of the mutants, including *cap2Δ/Δ*, showed a significant growth defect in medium containing excess iron (Fig. 1B).

**FIG 2:**
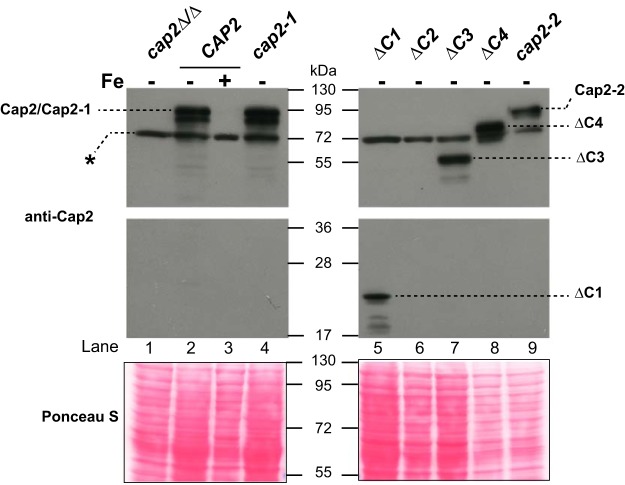
The C-terminal deletion mutant protein is expressed in iron-depleted medium. Whole-cell extracts from *cap2Δ/Δ* (lane 1), wild type (lanes 2 and 3), *cap2-1* (lane 4), *ΔC1* (lane 5), *ΔC2* (lane 6), *ΔC3* (lane 7), *ΔC4* (lane 8), and* cap2-2* (lane 9) strains were separated on 8 to 16% gradient SDS-polyacrylamide gels, transferred to nitrocellulose membranes, and probed with affinity-purified anti-Cap2 antibody. A 72-kDa nonspecific band that consistently appeared in all lanes as reported previously (9) is indicated by an asterisk.

To examine the expression of the various Cap2 mutant proteins, we next performed Western blot analysis. The isogenic wild-type and mutant strains were cultured in yeast extract-peptone-dextrose (YPD) plus 200 µM BPS and grown for 5 h, and whole-cell protein extracts were prepared and separated on 8 to 16% SDS-polyacrylamide gels. The blots were probed with affinity-purified anti-Cap2 antibody raised against the N-terminal 180 amino acids (6). Western blot results showed that, as reported previously (6), the wild-type Cap2 protein is induced in iron-depleted medium and repressed in iron-replete medium (Fig. 2). The Cap2-1 and Cap2-2 proteins bearing mutant bZIP and HAP4L domains, respectively, were also expressed at or close to the wild-type level under iron deprivation conditions (6). The Western blot data also showed that the ΔC1, ΔC3, and ΔC4 proteins were all expressed but to different extents in BPS medium (Fig. 2). However, in our repeated Western blot analyses, the Cap2 ΔC2 protein was not detected, indicating a likely instability of this mutant. Importantly, we note that the ΔC4 protein expression was robust at or close to the wild-type level (Fig. 2). Together, these data showed that the bZIP, HAP4L, and C-terminal ΔC4 mutant proteins were expressed at high levels under iron-depleted conditions.

### Cap2 C-terminal region and the bZIP-HAP4L domains are essential for iron-responsive gene expression.

**FIG 3:**
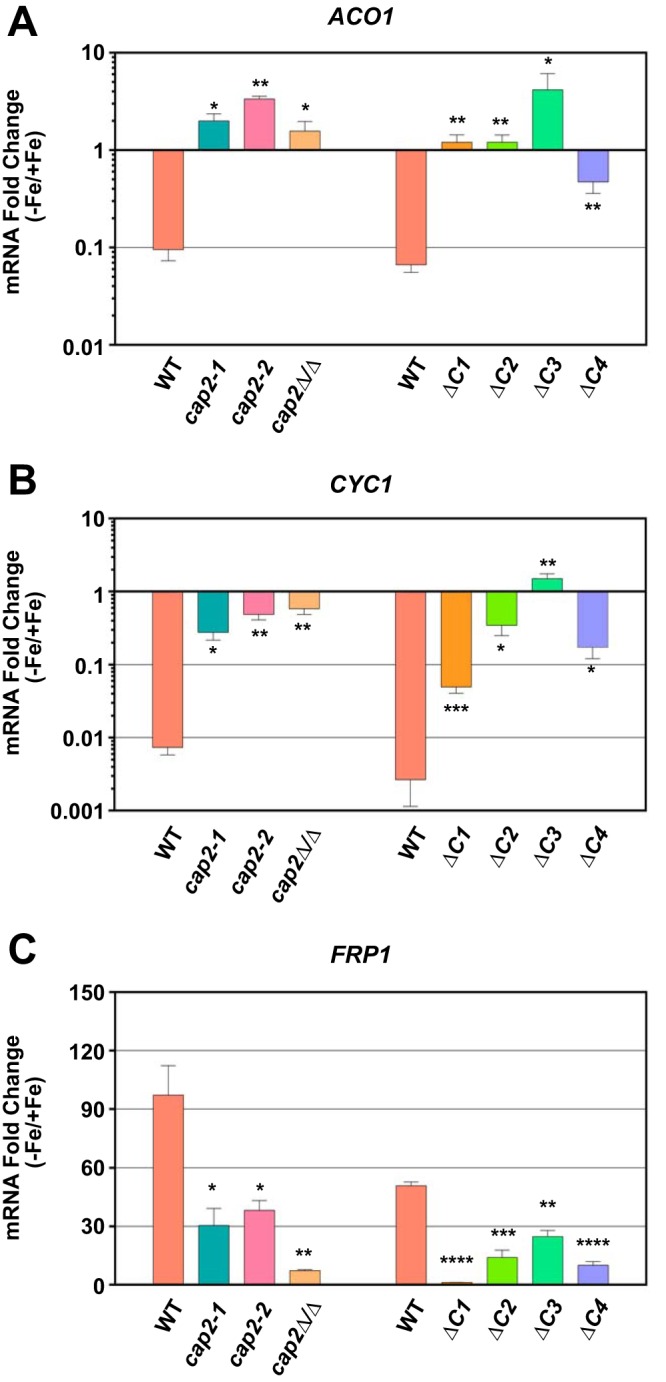
Transcriptional regulation of iron homeostasis genes is impaired in the bZIP, HAP4L, and C-terminal domain mutants. (A to C) qRT-PCR analysis of the expression of Cap2-repressed *ACO1* (A) and *CYC1* (B) mRNA levels and Cap2-induced *FRP1* mRNA levels (C) in wild-type and mutant strains. Fold change was calculated from three independent experiments, and Student’s *t* test was conducted to determine statistical significance. The values for mutants that are significantly different from the value for the wild type are indicated by asterisks as follows: *, *P* ≤ 0.05; **, *P* ≤ 0.01; ***, *P* ≤ 0.001; ****, *P* ≤ 0.0001.

Previous studies showed that deletion of *CAP2* impaired the activation of ferric reductase gene *FRP1* expression, and in contrast, derepressed the expression of *ACO1* and *CYC1* genes under Fe-depleted conditions (6–8). Therefore, we tested the various mutants for expression of iron homeostasis genes. Toward this end, all strains were cultured for 5 h in medium containing either BPS (Fe-depleted) or FAS (Fe-replete), total RNA was isolated, cDNA was prepared, and quantitative reverse transcription-PCR (qRT-PCR) was performed as described previously (6, 8). The qRT-PCR data showed that in the wild-type strain, *ACO1* and *CYC1* mRNA levels were highly downregulated, while the *FRP1* mRNA was upregulated in medium containing BPS compared to medium containing FAS (Fig. 3A to C). The *cap2* deletion, however, led to the derepression of *ACO1* and *CYC1* mRNA levels, and the *FRP1* mRNA level was significantly reduced (Fig. 3A to C) as reported previously (6–8). Furthermore, we observed significant derepression of *ACO1* and *CYC1* mRNA levels and reduction of the *FRP1* mRNA level in the bZIP domain mutant strain as well as in the HAP4L domain mutant strain (Fig. 3A to C), in agreement with the growth defect seen for *cap2-1* and *cap2-2* strains in medium containing BPS (Fig. 1B).

Next, we analyzed the mRNA levels in the C-terminal truncation mutant strains *ΔC1*, *ΔC2*, *ΔC3*, and *ΔC4* in medium containing BPS (−Fe) versus medium containing FAS (+Fe). Whereas *ACO1* and *CYC1* expression was depressed in the mutant strains compared to the wild-type strain, *FRP1* expression was impaired in each of the four mutant strains (Fig. 3A to C). Thus, taken together, the qRT-PCR data showed that each of the bZIP, HAP4L, and C-terminal conserved domains in Cap2 are required for the control of iron homeostasis gene expression under Fe-depleted conditions.

### Promoter targeting of Cap2 is critically dependent on the conserved domains.

**FIG 4:**
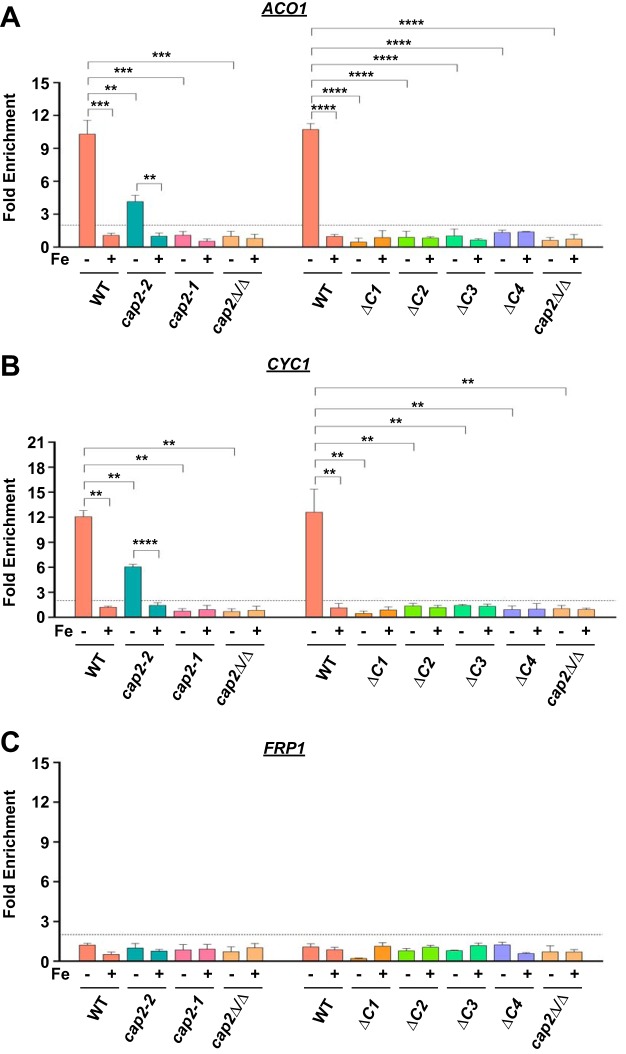
Promoter occupancy of Cap2 requires the bZIP, HAP4L, and C-terminal conserved domains. (A and B) The deletion of either *ΔC1*, *ΔC2*, *ΔC3*, and *ΔC4* or mutation in bZIP domain (*cap2-1*) or deletion of HAP4L domain (*cap2-2*) abolished Cap2 recruitment at *ACO1* (A) and *CYC1* (B) promoters compared to the wild-type control under iron-depleted conditions. (C) Cap2 is not recruited to the *FRP1* promoter in the WT and mutants. The values that are significantly different are indicated by bars and asterisks as follows: **, *P* ≤ 0.01; ***, *P* ≤ 0.001; ****, *P* ≤ 0.0001.

To address how the Cap2 domains control iron homeostasis gene expression, we conducted chromatin immunoprecipitation (ChIP) assays. Genome-wide ChIP analysis showed that Cap2 was recruited to *ACO1* and *CYC1* promoters but not to the *FRP1* promoter (8). Hence, we performed ChIP assays to examine the occupancy of Cap2 mutant variants at *ACO1*, *CYC1*, and *FRP1* promoters in chromatin extracts prepared from strains cultured in iron-depleted (−Fe) or iron-replete (+Fe) medium. The ChIP assays revealed that the wild-type Cap2 was recruited to *ACO1* (~10.3-fold; Fig. 4A) and *CYC1* (~12-fold; Fig. 4B) promoters, but not to the *FRP1* promoter (Fig. 4C) under iron-depleted conditions. The Cap2 occupancy at *ACO1* and *CYC1* promoters was either completely lost in the bZIP mutant (*cap2-1*) or significantly impaired in the HAP4L (*cap2-2*) mutant strains (Fig. 4A and B). Thus, the bZIP domain is essential for Cap2 recruitment at the *ACO1* and *CYC1* promoters.

Next, we examined the recruitment of *ΔC1*, *ΔC2*, *ΔC3*, and *ΔC4* Cap2 mutants at the same promoters. The Cap2 mutants, like the wild-type Cap2, were not recruited to the *FRP1* promoter (Fig. 4C). However, rather unexpectedly, the occupancy of each of the C-terminal mutants was lost at the *ACO1* and *CYC1* promoters (Fig. 4A and B). Because the expression levels of the ΔC1 and ΔC2 proteins seem to be compromised (Fig. 2), their recruitment defect cannot be established. As ΔC3 and ΔC4 proteins were expressed at high levels, we conclude that their recruitment defect was a consequence of the mutations. Furthermore, as the ΔC4 protein carried a shorter truncation compared to the ΔC3 protein and yet failed to be recruited, we conclude that the C-terminal 54 amino acids is essential for Cap2 recruitment. Together, the ChIP data showed that the bZIP, HAP4L, and C-terminal conserved domains are critical for recruitment of Cap2 to promoter *in vivo*.

## DISCUSSION

Iron homeostasis gene expression in C. albicans is controlled by the transcriptional regulators Cap2/Hap43, the trimeric HAP complex, Sef1, and Sfu1 (reviewed in references 15 and 16). While the HAP complex, represented by the Hap5 subunit, is recruited to both induced as well as repressed gene promoters under iron-deficient conditions (6), Cap2 is recruited to the repressed promoters such as *ACO1* and *CYC1*, and Sef1 is recruited to induced promoters (8). Moreover, Cap2 and the HAP complex interact to form the Cap2-HAP complex (6). Cap2 is a large protein comprised of the evolutionarily conserved N-terminal bZIP domain and HAP4L domain, and several conserved segments in its C-terminal region (6). Point mutations within the DNA-binding residues in the bZIP domain and a deletion of the 17-amino-acid HAP4L domain abrogated Cap2 function (6). This bipartite bZIP-HAP4L domain is evolutionarily conserved in dikaryal fungal genomes with the exception of the *Saccharomyces* genomes.

We constructed and analyzed the activity of four Cap2 deletion mutants Δ*C1* to Δ*C4* bearing progressive truncations from the C-terminal region and found that all four mutants were defective for growth and transcriptional regulation in medium containing BPS. Given that the Δ*C4* mutant, bearing the shortest deletion that removed Cap2 amino acid residues 497 to 634, had all the phenotypes shown by Δ*C1* to Δ*C3* mutants, suggested that the loss of the extreme C terminus caused the defect in each mutant. Indeed, the Cap2 amino acid sequences from 13 closely related *Candida* clade genomes showed extremely high sequence conservation in this region, underscoring its significance.

While this work was in completion, a series of Cap2 deletion mutants were reported, including a large deletion spanning the entire bZIP domain and the Hap4L domain in the N-terminal region, and several deletion mutants spanning the C-terminal region (13). These N-terminal and C-terminal deletion mutants were deficient for growth and gene expression in medium containing hemoglobin as the sole iron source. Targeted deletion of the specific C-terminal regions that removed the Cys-rich regions did not impair Cap2 function. However, the expression level of each mutant protein and the molecular defect associated with each mutant were not known. Our analysis adds to the understanding of Cap2 function by distinguishing between defects in protein stability and DNA binding that ultimately impact functional activity.

The *CAP2* ortholog *HapX* gene is required for the iron starvation response in A. nidulans (11), A. fumigatus (17), and C. neoformans (9) and mediates repression of iron utilization genes and activation of iron uptake genes and siderophore biosynthesis genes. While HapX is required for resistance to excess iron in *Aspergillus* spp. (12), Cap2 is not required for resistance to excess iron in C. albicans (Fig. 1B). The N-terminal bZIP and HAP4L domains of HapX are required for transcriptional induction of genes involved in iron uptake and siderophore biosynthesis under iron deprivation conditions in A. fumigatus (17). However, the HapX C-terminal Cys-rich regions, termed CRR domains, were dispensable for the iron starvation response (12). Thus, the orthologous, evolutionarily conserved transcription factors *Aspergillus* HapX and *Candida* Cap2 have diverged in their functions.

Our analyses have uncovered three major determinants of Cap2 promoter recruitment. First, the HAP4L mutation, bearing the deletion of the Hap5 interaction domain, impaired the promoter occupancy of the HAP4L mutant, indicating an essential *in vivo* requirement of the interaction of Cap2 HAP4L domain with the trimeric Hap2-Hap3-Hap5 complex (6). In this context, Hap5 is recruited to promoters *in vivo* under iron deprivation conditions (6), and Hap5 promoter binding requires the CCAAT box *in vitro* (5). Second, the critical amino acid residues in the bZIP DNA-binding domain abrogated Cap2 recruitment. Finally, the C-terminal 54-amino-acid region was essential for Cap2 function and promoter recruitment. These results cannot distinguish between whether each of the three protein domains examined here can act independently or whether they act synergistically in some way to stimulate Cap2 transcriptional activity. The bZIP, HAP4L, and C-terminal regions are highly conserved in Cap2 and HapX orthologs, suggesting an evolutionary conservation of the promoter targeting function for the Cap2 domains. Thus, by employing the three Cap2 domains for transcriptional activity and promoter recruitment functions, fungal pathogens have developed exquisite control of the activity of this critical transcriptional regulator for iron homeostasis regulation.

## MATERIALS AND METHODS

### Growth conditions.

Candida albicans strains were cultured in synthetic complete limited-iron medium (SC-LIM) as described previously (6). For iron-depleted medium, bathophenanthrolinedisulfonic acid (BPS) was added to a final concentration of 100 µM (SC-LIM) or 200 µM (yeast extract-peptone-dextrose [YPD]). For iron-replete medium, ferrous ammonium sulfate (FAS) was added to a final concentration of 100 µM (SC-LIM) or 200 µM (YPD) to the respective iron-depleted medium. Strains were cultured in YPD or SC-LIM medium for Western blot and ChIP experiments, respectively, and grown for 14 to 16 h at 30°C and diluted to an initial optical density at 600 nm (OD_600_) of 0.25 (wild type) or 0.5 (mutants) under iron-depleted and -replete conditions, grown for 5 h, and harvested.

### Construction of strains and plasmids.

The list of plasmids, strains, and oligonucleotides used in this study are provided in Tables S1 to S3 at https://www.jnu.ac.in/Faculty/natarajan/data.htm. The *CAP2* plasmids bearing C-terminal truncation mutations were constructed by PCR amplification of *CAP2* regions from pRC20 (6) using oligonucleotide pairs ONC45/ONC10 (*ΔC1* mutant), ONC45/ONC321 (*ΔC2* mutant), ONC45/ONC322 (*ΔC3* mutant), and ONC45/ONC323 (*ΔC4* mutant) and cloned as follows. The amplicon containing *ΔC1* was digested with BsmBI, blunt ended, and ligated to Tth111I-BsmBI-digested plasmid pRC20, and pNA3 was obtained. The amplicons bearing *ΔC2*, *ΔC3*, and *ΔC4* sequences and the plasmid pRC20 were sequentially digested with TthIII1 and BsmBI and ligated together, yielding plasmids pNA18, pNA26, and pNA22, respectively. All constructs were sequenced in its entirety using oligonucleotides ONC45, ONC56, and ONC57 covering the insert from positions −12 to +1905 with reference to the *CAP2* ATG position. While *ΔC2* and *ΔC3* inserts matched the wild-type sequence, the *ΔC1* and *ΔC4* inserts contained substitution mutations leading to double amino acid substitutions Tyr45Ser and Pro47Leu changes. The *ΔC4* insert also contained two other fortuitous nucleotide substitutions, viz., the silent mutation G774A, and the T1278C mutation leading to the Pro427Ser substitution. The plasmid constructs were digested with StuI and integrated at the *RPS1* locus in the *cap2Δ/Δ* mutant strain (RPC75), the correct integration was confirmed by PCR, and strains RPY468, RPY527, RPY535 and RPY568 were obtained. The *CAP2-1* (RPC286) and *CAP2-2* (RPC326) strains have been described previously (6).

### Whole-cell extract preparation and Western blotting.

The cell pellets were resuspended in chilled Winston buffer (40 mM HEPES-NaOH [pH 7.5], 350 mM NaCl, 10% glycerol, 0.1% Tween 20) (18) with added protease inhibitors (2.5 µg/ml aprotinin, 2 mM benzamidine, 1 mM dithiothreitol, 2 µg/ml leupeptin, 2 µg/ml pepstatin, 100 µM phenylmethylsulfonyl fluoride [PMSF], 10 µg/ml tosylsulfonyl phenylalanyl chloromethyl ketone [TPCK], 10 µg/ml *N*α-*p*-tosyl-l-lysine chloromethyl ketone [TLCK]) and vortexed in the presence of glass beads. The lysates were cleared by centrifugation at 13,000 rpm for 15 min at 4°C. Cell lysates were resolved on SDS-polyacrylamide gels, transferred to nitrocellulose membranes, probed with anti-Cap2 antibody, treated with ECL-Prime Western blot detection reagent (GE Healthcare), and exposed to X-ray film.

### RNA analysis.

Total RNA was isolated from C. albicans cells, and cDNA was synthesized as described previously (6). Real-time quantitative reverse transcription-PCR (qRT-PCR) was carried out in an Applied Biosystems Fast 7500 or Bio-Rad CFX96 real-time PCR system using gene-specific primers, and differential expression was calculated by the comparative threshold cycle (*C*_*T*_) method (19). The *SCR1* RNA, an RNA polymerase III transcript, was used as the endogenous control (6).

### ChIP assay.

Chromatin immunoprecipitation (ChIP) assays were conducted as described previously (6). Briefly, the chromatin extracts were prepared by shearing in a Bioruptor (model UCD 300; Diagenode) for 35 cycles with 1 cycle consisting of 30 s on and 30 s off. About 2 µl of affinity-purified Cap2 antibody was bound to 30 µl of protein G FF beads (GE Healthcare) and chromatin extracts equivalent to ~35 OD_600_ equivalent of cells from control and experimental samples were immunoprecipitated for 4 h at 4°C. The input DNA and immunoprecipitated (IP) DNA were de-cross-linked, followed by phenol-chloroform extraction. The total DNA (input; 1:10,000) and IP DNA (1:5) were analyzed by qRT-PCR for enrichment of specific regions of interest as well as for the control nonspecific region (ca21chr1_1573500–1574000). Enrichment was calculated for target regions and for the control nonspecific region (ca21chr1_1573500–1574000), and specific enrichment was calculated with respect to input total chromatin. statistical significance was determined by the Student’s *t* test in GraphPad Prism 6. A *P* value of ≤0.05 was considered statistically significant.

### Data availability.

All data are included in the paper and in the supplemental files at https://www.jnu.ac.in/Faculty/natarajan/data.htm.
